# More than a Bundle? Developing Adaptive Guidance for Task Selection in an Online, Semantic-Based Cognitive Stimulation Program

**DOI:** 10.3390/brainsci15040419

**Published:** 2025-04-20

**Authors:** Ana Rita Batista, Vasiliki Folia, Susana Silva

**Affiliations:** 1 Center for Psychology, Faculty of Psychology and Educational Sciences, Psychology Department, University of Porto, Rua Alfredo Allen, s/n, 4200-135 Porto, Portugal; 2 Laboratory of Neuropsychology and Behavioral Neuroscience, School of Psychology, Aristotle University of Thessaloniki, University Campus, 546 26 Thessaloniki, Greece

**Keywords:** cognitive stimulation, cognitive training, adaptive advice, task selection, tailor-made, web-based

## Abstract

Background: Cognitive stimulation programs typically consist of task collections (“bundles”) designed to cover various aspects of a cognitive domain and/or sustain user engagement. However, task order is often overlooked, despite variations in difficulty based on structure or mode of implementation. This study examined users’ performance accuracy across the eight tasks that comprise the BOX semantic-based program, adapted for the Cerup/CQ online platforms. Our ultimate goal was to map the tasks onto increasing levels of challenge within thematic clusters to provide guidance for personalized task selection. Methods: After adapting the program into Portuguese using original materials based on BOX task descriptions, we made Cerup and CQ (which share the same content but have different layouts) available as free web-based tools. Participants, primarily older adults without dementia, were invited to use these platforms for cognitive stimulation. We analyzed accuracy data as a function of activity-related characteristics (complexity scores, sentence- vs. word-level) as well as participants’ spontaneous task selection. Results: Task characteristics influenced performance accuracy, indicating different levels of challenge across activities. However, spontaneous task selection did not follow any discernible pattern beyond the spatial contiguity of activity buttons, which was unrelated to participants’ likelihood of success. Based on these findings, we defined optimal navigation paths for the eight tasks. Conclusions: Challenge-based, active guidance for task selection appears justified and necessary within the BOX/Cerup/CQ programs. Additionally, the method we developed may help other programs enhance user experience and optimize task progression.

## 1. Introduction

Semantics is the study of meaning, and linguistic semantics specifically focuses on how meaning is conveyed within the language system, as opposed to other forms of communication, e.g., facial expressions. Linguistic meaning exists at the level of individual words (word-level or lexical semantics), but also depends on the sequential arrangement of words into sentences according to the rules of a given grammar (sentence-level or syntactic semantics) [1,2,3].

Deficits in linguistic semantics are associated with various neurological conditions, primarily degenerative, such as semantic dementia, posterior cortical atrophy [4], frontotemporal dementia [5], aphasia, and Alzheimer’s disease [6]. Production difficulties, particularly lexical retrieval, are pervasive in degenerative diseases like Alzheimer’s [6,7,8]. In semantic dementia, patients exhibit impairments in comprehension (e.g., lexical decision) alongside more apparent production difficulties, such as lexical retrieval deficits [9,10]. Similar issues arise in aphasia, where different subtypes affect either comprehension (e.g., global aphasia) or production (e.g., non-fluent aphasia) [11,12]. Although research and neuropsychological practice often focus on the most basic semantic level- the word level- sentence-level semantics is also compromised in these clinical conditions, as word-level processing is a prerequisite for sentence comprehension. Given the crucial role of semantic processing for basic social functioning and the susceptibility of the semantic system to degeneration and/or brain lesions, the need for methods and tools to rehabilitate or preserve semantic abilities is well justified. The so-called “lexical semantic therapies” (LS) [13,14] were developed as semantic exercises aimed at remediating linguistic semantic deficits and aid patients in rehabilitation. To enhance clarity, we refer to these as “semantic therapies”, as the prefix “lexical” may imply a restriction to word-level semantics.

The literature presents mixed findings on linguistic semantic processing in healthy aging. Some studies report no significant differences between younger and older adults (e.g., [15,16,17]), while others highlight word-level difficulties in older participants, particularly in production accuracy and speed [18,19,20,21,22]. Comprehension difficulties appear to be absent, and occasional low performance under certain conditions may be attributed to age-related decline. However, semantic methods and exercises not only enhance semantic abilities, but also improve general language skills, such as phonological abilities [6,23,24,25]. Moreover, semantic exercises may have a positive impact on general cognition. For instance, studies on patients with early Alzheimer’s disease have shown that semantic therapy can improve episodic memory [25], working memory, and processing speed [26]. Furthermore, the extensive interconnections between linguistic and non-linguistic skills (notably executive functions) suggest that training in one domain may benefit the other [23]. Specifically, semantic skills are strongly associated with inhibition [27] and working memory [28]. Moreover, sentence-level semantics has a privileged connection with memory updating, both at the levels of sentence comprehension [29] and production [30]. In summary, semantic deficits—particularly those related to comprehension—are not necessarily inherent to healthy aging. However, semantic training appears to have positive effects in other domains. From this perspective, employing semantic tasks for cognitive stimulation (focusing on training and prevention, as opposed to rehabilitation) could be a logical approach.

Despite the potential of semantic training as a cognitive stimulation tool, systematic reviews of available programs indicate limited investment in this area [31,32]. One exemption is the instrumental use of categorization—a memory training intervention—to enhance memorization, though this is generally regarded as a compensatory strategy [33]. The instrumental use of categorization refers to the deliberate strategy of organizing information into meaningful groups/categories, sharing the same characteristics. This technique enhances memory retention, recall, and general cognitive efficiency, since it reduces cognitive load and creates associative links between items [34]. This is particularly valuable for older adults due to possible age-related cognitive declines they might face [35,36]. Consequently, categorization is considered a compensatory strategy, because it is often employed to balance limitations in working memory or attention, especially in individuals with cognitive impairments or in older adults.

To address this gap and promote LS stimulation in healthy and mildly impaired aging, we adapted the BOX linguistic semantic program (originally developed by [22] to address naming and word retrieval difficulties in aphasia, and used in [25,26]. Its focus is lexical semantic processing, operating at the lexical (e.g., individual word meanings) and the sentence levels (e.g., understanding semantics in context). It provides structured practice, and tasks can be adjusted to fit a patient’s specific profile. It consists of eight components: (1) semantic categories, (2) syntagmatic and paradigmatic relationships, (3) semantic gradation, (4) adjectives and exclamations, (5) part–whole relationships, (6) anomalous sentences, (7) semantic definitions, and (8) semantic context. The exercises are presented in multiple-choice or right/wrong format. Patients are asked to confirm or deny semantic relationships between content words, either in isolation or within larger sentence contexts. Moreover, the level of difficulty is adjusted through word properties (i.e., imageability, frequency, word length, abstractness), distractor count (i.e., more distractors increase task complexity), semantic relatedness (i.e., unrelated distractors for easier tasks, semantically related ones for harder tasks), and ambiguity (i.e., use of ambiguous words to challenge the patient’s ability to process multiple meanings simultaneously) [37,38]. Regarding its efficacy and design quality, the BOX program is well regarded for its structured and theoretically grounded design [39]. Visch-Brink et al. [22] and subsequent studies [40] have shown positive outcomes in some patients, especially those with mild to moderate aphasia and preserved written comprehension. However, evidence is still limited, and results have not yet been widely replicated or tested in other languages [41]. Additionally, direct comparison between BOX and other forms of rehabilitation approaches is scarce (e.g., [42]).

Therefore, lexical semantics therapies (such as the BOX therapy) are more specific to language therapy, particularly in aphasia, belonging to the broader term of semantic-based programs. Such programs so far have mainly been used for cognitive rehabilitation interventions. Consequently, the proposed training activities in this study fall into lexical semantics therapy since they are adapted from the BOX therapy. However, in this paper we explore the BOX program above and out of the frame of aphasia. Ultimately, we want to explore the possibility of a semantic-based cognitive rehabilitation tool being used for semantic-based cognitive stimulation interventions.

We made BOX freely available on an online platform, Cerup (https://estimulacerup.wixsite.com/website; accessed on 18 April 2025), for independent use or with the assistance of a facilitator (e.g., practitioners or others). Subsequently, we developed an expanded version of Cerup, the “Question Club” platform (www.clubedasquestoes.pt). This new platform integrates Cerup as a “play” mode while introducing a new feature—a “contribute” mode—where participants can create multiple-choice questions for other participants, becoming content creators and enriching the platform.

**Figure 1:**
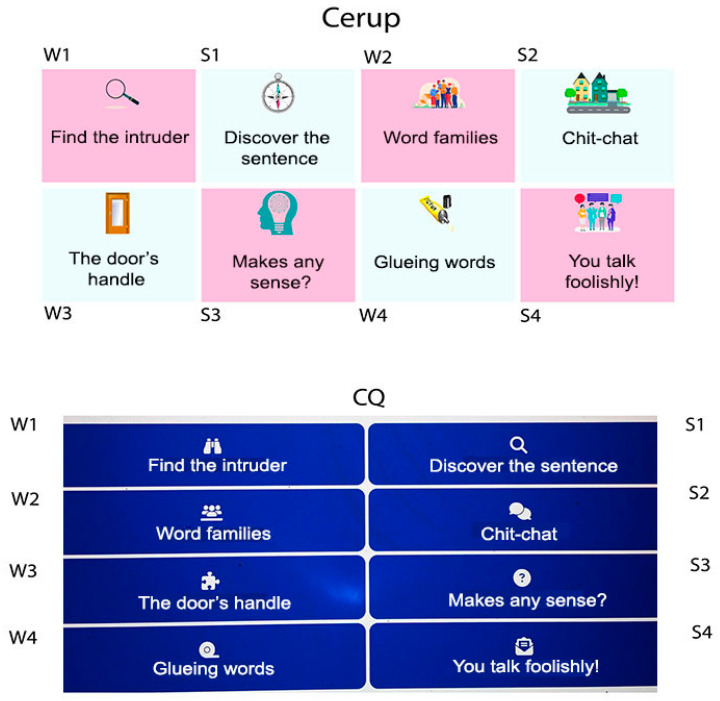
Layout of Cerup and CQ menus. The labels W and S are not displayed on the websites. In the Cerup layout, the three within-task difficulty levels are directly accessible in the menu (in CQ, they appear after clicking on the task name).

Cerup and the play mode of Question Club (CQ, hereafter) contain the same set of eight semantic activities, each organized into blocks of multiple-choice questions (Figure 1). Users receive feedback after each question (indicating correct or incorrect responses) and block (providing the number of hits and average response time). Both platforms are free, web-based tools accessible to anyone with an internet connection and a computer, tablet, or smartphone. Free online platforms enable users to participate remotely and at no cost, making cognitive stimulation more accessible to older adults. However, accessibility does not come without risks. First, online activities present challenges for both users and practitioners. Older users may experience age-related limitations, such as hearing and/or vision impairments or a lack of confidence in using computer-based technology, while practitioners may face additional demands, including the need for initial training and technical proficiency (see [43]). Nevertheless, research indicates that computer-based technology is well accepted by patients undergoing rehabilitation [44,45], suggesting that the same may hold true for healthy older adults. Critically, the BOX program, which we adapted, was tested in both face-to-face and computer-based remote modalities, yielding similar outcomes [26]. A second risk associated with free platforms is that users, or even practitioners, may not fully maximize the program’s benefits when adaptive guidance is not optimized. Adaptive guidance refers to the provision of recommendations on how to use the program in a way that best aligns the individual’s needs. This may include offering feedback or addressing the selection of specific tasks and/or difficulty levels to enhance motivation, engagement, and progress for a particular individual or group [46]. The importance of personalizing cognitive stimulation through adaptive guidance has been increasingly recognized, as opposed to the traditional “one-size-fits-all” approach [47]. Tailor-made interventions have demonstrated advantages [48] and, to some extent, have been promoted by commercially available computerized programs. A 2020 systematic review examining 11 computerized tools for cognitive stimulation, training, and rehabilitation [49] found that all but one allowed users to select a difficulty level within each task, and all provided feedback. In terms of task selection, 10 out of 11 tools based their choices on broad cognitive domains (e.g., memory, calculus, language, etc.), while the remaining tool focused on a single cognitive domain.

Regarding the BOX program, its original version incorporated some adaptive guidance, though in a limited way. In addition to offering feedback, each of the eight BOX activities was designed with three difficulty levels (easy, medium, difficult), providing some level of guidance. However, to our knowledge, the possibility of personalizing the user experience based on the specific characteristics of each activity or the social context of administration has not yet been considered. While semantic-based training is a relatively narrow domain, semantic tasks can serve different functions [50], which may also apply to BOX activities. Some tasks go beyond simple meaning extraction, targeting other semantic domains such as categorization or part–whole relationships, while one activity focuses on conversational skills and linguistic pragmatics. Although these differences may be relevant—both because they represent distinct cognitive domains and potentially different levels of difficulty—they have not, to our knowledge, been considered in the context of adaptive guidance. The BOX program also contains other task-related potential sources of difficulty. Activities vary in unit size, meaning that users may need to process a single linguistic segment (single) or compare two segments (composite) to determine the correct response. Additionally, tasks differ in the number of cognitive steps required to arrive at the correct response. None of these potential sources of difficulty have been highlighted in the BOX literature (e.g., [40]). The same applies to the word- vs. sentence-meaning duality, which is embedded in the program but has not been explicitly addressed. Beyond providing feedback after each response and block, our adapted online versions of BOX—Cerup/CQ—have not made significant progress in terms of adaptive guidance. Like the original version, these platforms currently present no more than a bundle of activities that complement one another and provide variety.

The goal of the current study was to design evidence-based adaptive guidance for task selection in the Cerup/CQ online platforms, based on the hypothesis that their eight activities present different levels of challenge, which users may not be aware of. Specifically, we sought to define what we termed “optimal paths”, i.e., structured ways of navigating the platform in a progressive manner that might favor a sense of accomplishment, and this, in turn, may enhance training effectiveness. These optimal paths were built on two key principles: one was grouping activities into sets (referred to as tours) when they shared similar cognitive targets, and the other sequencing activities within and across tours from the least to the most challenging. To test the hypothesis that (1) activities differ in difficulty but (2) participants do not naturally optimize their navigation, we took the following steps. To test (1) and (2), we (1) examined participants’ accuracy across tasks (lower accuracy as increased challenge) and (2) compared participants navigation paths (task selection) with both accuracy levels and predefined complexity levels.

The performance accuracy of Cerup and CQ online users was examined in relation to three different task characteristics: activity (8 levels), word- vs. sentence-related tasks (2 levels), and complexity (4 levels). Complexity scores were derived from a predefined map of hypothesized activity-specific features. Cerup data were collected as part of a small-scale preliminary usability study (also included here), where participants rated the difficulty and clarity of instructions after playing their freely chosen activity. In contrast, CQ data came from unrestricted, game-like participation by registered users. Due to these and other cross-platform differences, we inspected platform effects to control for potential confounds. In both cases, participants used the platform freely, without guidance on task selection. In addition to task-related factors, we analyzed the effects of the context of administration—specifically, user type (individual vs. group) and different population types within groups—since these variables were part of the collected dataset. It is important to note that our primary focus was the relative difficulty of each of the eight activities rather than users’ overall performance, although the accuracy data we examined provided some insights into this aspect. As we hope to have made clear, assessing the cognitive impact of the program was not within the scope of this study, as such an investigation would require a different research design.

## 2. Materials and Methods

### 2.1. Participants

Since our goal was not to assess the impact of cognitive training, we excluded only adults diagnosed with dementia from data collection. Consequently, we included all individuals perceived by their caregivers as cognitively functional or mildly impaired. For users who engaged directly with the platform without assistance, we assumed that their cognitive function was at least minimally preserved, as they would otherwise be unable to use the platform independently. We imposed no specific requirements regarding the mode of administration (group vs. individual sessions) during recruitment, but most institutions chose the group session mode (all, in the case of Cerup).

To collect data on the Cerup platform, we reached out to 112 nursing homes and day centers nationwide, inviting them to participate in a usability study by accessing the website. Fifteen users participated in the study. Fourteen were individual users aged 29–90 (21% male, 42% professionally active) who did not report any health conditions that could affect cognitive functioning. The fifteenth user was a group of multiple sclerosis (MS) patients aged 37–53 years (15% males, non-active), attending an association, where they provided their responses during group sessions led by an intern psychologist.

For CQ, we also contacted institutions that work with older adults, such as day centers and nursing homes, as well as some institutions dedicated to supporting individuals with cognitive deficits, regardless of age. Providing sociodemographic data was optional for those who created an account. During the first year of activity, 37 accounts were created, of which only 19 (13 institutions and 6 individuals) actively used the platform in either play and/or contribute mode. Among the individual users, five were female and one was male. Only four reported their age (ranging from 35 to 59 years). Among the individual users, two were active, three were inactive and one did not provide any information. In total, we collected responses from 34 users (Table 1), comprising 20 individuals and 14 groups.

### 2.2. Component Activities

Based on descriptions and examples from the literature, we freely adapted the eight activities that compose the BOX lexical semantic rehabilitation program [22,51] for European Portuguese. Each activity was available at three different levels of difficulty—easy, medium, and difficult—depending on predefined characteristics (see below). Four activities focused on single-word meaning (W1–W4), while the other four required semantic integration at the sentence- or text-level (S1–S4).

Table 2 categorizes each activity according to four complexity indices. The first index indicates whether the linguistic target is a *single* (1) *or a composite unit* (2), as in W3, where users engage with two analogous sentences. The second index, *number of steps*, describes the minimum number of operations required to reach the correct response (cardinal). The *additional cognitive processes* parameter specifies whether users must perform tasks beyond extracting meaning (0 = no, 1 = yes). The fourth complexity index evaluates *response uncertainty*, *which reflects* whether participants remained uncertain about their success at these additional processing levels (0 = no, 1 = yes). By summing the assigned scores, we obtained a *global complexity score*, which was used as an independent variable in analyzing users’ spontaneous navigation and performance. Below, we describe the structure of each activity.

W1—FIND THE INTRUDER: Originally titled “Semantic Categories”, this activity presents lists of five semantically related words mixed with one word from a different category—the “intruder”. Participants are asked to identify the intruder among the six options. For instance, in the list consisting of “skirt, socks, shirt, curtain, coat, trousers”, most words refer to clothing items, making “curtain” the intruder. The pre-assigned difficulty level decreased with word frequency, concreteness, the degree of common knowledge (as opposed to jargon or specialized terms), and the semantic unrelatedness of the intruder.

The task required participants to carry out two interdependent operations: (1) identify the dominant category/object type (e.g., recognizing that most items in the list are clothing) and (2) find the option outside that category. If step (2) could not be completed, step (1) had to be reconsidered. In addition to understanding word meanings, participants needed to activate the hierarchical representations inherent in categorization (e.g., recognizing that both skirt and shirt belong to the broader category of clothing). Notably, participants’ guess for the dominant category—an intermediate step in the task—remained uncertain, as it was not explicitly listed among the response options; only the intruder was provided.

W2—WORD FAMILIES: Closely related to semantic categories, this task was originally named “Semantic Classification”. It involved presenting participants with a list of words that belonged to a specific but undefined category. Participants were then given two category names as response options, one correct and one incorrect. For example, they might see the words: tulip, daisy, rose, carnation, sunflower, orchid, lilium, with “flowers” and “vegetables” as response options. The correct answer would be “flowers”. As in W1, the difficulty level decreased with word frequency, concreteness, and the extent of common knowledge (as opposed to jargon or specialized knowledge). In this case, the semantic unrelatedness of the incorrect option made the question easier.

To complete the task, participants could arrive at the correct answer in two ways. First, they could identify the category as they saw the items (e.g., looks like flowers) and then check if the corresponding category was listed as an option (yes). Alternatively, they could start by examining the two response options and then review the items (one would be enough) to eliminate one of them (e.g., tulip is a flower, not a vegetable). Although categorization processes were also involved in this task, only one basic step was required: recognizing the correct category from the two options. This made W2 potentially less complex than W1.

W3—THE DOOR’S HANDLE: In this task, originally designated as “Part–Whole Relationships”, participants were required to identify the missing word in an analogy structured as “x is to X as y is to Y”, where x and y represent parts, and X and Y represent the corresponding wholes, or vice-versa. The missing word could be any of these four, and participants had four answer choices from which to select the correct one. For example, given the sentence “Screen is to laptop as wall is to …”, with the options “door, ceiling, concrete, room”, the correct answer would be “room”. At the easy level, only the final word was missing. At the medium and difficult levels, other words within the analogy could also be missing.

To successfully accomplish the task, participants needed to (1) abstract the part–whole relationship from the complete “is to” construction, (2) identify whether the missing element was a part or a whole within the incomplete analogy construction, and (3) find the word that instantiated the missing role from the given answer choices. Responses to step (1)—an intermediate step—remained uncertain, as they were not included in the response options.

W4—GLUING WORDS: Originally named “Compound Words”, this task required participants to identify valid compound words formed by combining a given root word with other words. For example, given the prefix “well-” participants were presented with four possible compounds, only one of which was correct (e.g., “well-educated, well-cat, well-umbrella, well-eat”, with the first option being correct). The difficulty increased based on word frequency and potential unfamiliarity (jargon, or specialized terminology).

To successfully complete the task, participants could make lexical decisions by determining whether each option was a real word.

S1—DISCOVER THE SENTENCE: This sentence-level semantic task, previously referred to as “Syntagmatic and Paradigmatic Relationships”, required participants to complete a sentence by selecting the most appropriate word or word group from four options. For instance, given the incomplete sentence “Paul ate…” with the options “a chair, a story, an ice cream, the door”, the correct answer would be “an ice cream”. Incorrect choices could be semantically incongruent (as in this example) or grammatically incorrect (e.g., “Paul ate …jumped”, where two verbs follow each other). In the latter case, semantic incongruence also naturally occurs. Task difficulty varied based on sentence complexity (simple, coordinate, or subordinate sentences).

To complete the task, participants could follow a process similar to W2. They could either (1) identify key characteristics of the missing part as they read the incomplete sentence (e.g., “Paul ate…” suggests food) or (2) evaluate the semantic congruence of the sentence with each possible answer.

S2—CHIT–CHAT: This task addressed sentence-level semantics in conversational contexts. Participants read a transcription of a speaker’s statement (e.g., “John got himself into trouble again”) and chose the most adequate listener response from four options (e.g., “How happy he is!; He is so conflictual!; Very sensitive!; How vain!”, with the second option being the correct one). The original task name referred to the use of adjectives and exclamations in responses. Task difficulty increased with increasing syntactic complexity, and at the third level, responses included options with analogies and proverbs, requiring greater abstraction.

To accomplish the task, participants could (1) extract meaning from the speakers’ statements and apply theory of mind [52] skills to infer their stance or judgment (e.g., recognizing that the speaker is implicitly condemning John). Afterward, participants could (2) either generate an appropriate listener response and match it to the given options (e.g., agreeing with the speaker by selecting “How conflictual!”) or evaluate each response option individually. However, inferences regarding the speaker’s intent (theory of mind) remained uncertain.

S3—MAKES ANY SENSE? Originally named “Anomalous Sentences”, this task required participants to assess the semantic coherence of sentences. In the first level, participants simply judged sentences as either “correct” or “incorrect”. In the second, they identified the specific word that made the sentence anomalous, when applicable. In level 3, they selected a replacement word to restore the sentence’s meaning. At its easiest level, this task involved basic sentence-level semantic judgments, making it potentially simpler than S1. The medium and difficult levels introduced additional cognitive demands.

S4—YOU TALK FOOLISHLY! Previously titled “Semantic Context”, this task involved identifying semantic anomalies in longer text segments (ranging from two to five sentences). For instance, in the sequence “Tomorrow I will cut my hair. Nevertheless, the market is closed”, the second sentence is unrelated to the first, making the passage semantically incoherent. In level 1, participants selected the correct text from two options. Levels 2 and 3 presented increasingly longer texts, one at a time, requiring participants to judge whether each text was semantically coherent or incoherent.

### 2.3. Procedure

For Cerup, data collection was conducted online over two and a half months (19 April–4 June 2021). Since the provision of personal data was mandatory, we emphasized data confidentiality and anonymity, ensuring that no unnecessary personal information, such as contact details or names, was collected. The study was approved by the Ethics Committee of the Faculty of Psychology and Educational Sciences at the University of Porto, (authorization number: Ref.ª 2021/09-05b).

The eight activities, each with three difficulty levels, were structured into 24 different experiments using OpenSesame, version 3.3.9 Lentiform Loewenfeld. OpenSesame [53] is free software that allows online data collection once experiments are hosted on a server like Jatos [54]. At the time, online data collection was only possible using a computer (as opposed to a tablet or smartphone). To facilitate user access to the activities and supporting information (Supplementary), we created a website (https://estimulacerup.wixsite.com/play/atividades, accessed on 18 April 2025) where links to all 24 experiments were organized on a single page. In addition to a brief description of each activity, the website hosted an informed consent form, an overview of the project, and contact information. The activities were renamed (as described in the Section 2) and cover images (Figure 1) were added to enhance their appeal. Response modes were primarily based on single-click interactions. Navigation instructions were provided at every step, written in simple language to ensure clarity.

Upon accessing the website, participants were required to read the study information and provide their consent by selecting a checkbox. They were then directed to the main menu, which contained the links to the eight tasks across three difficulty levels, hosted on Jatos. Upon opening a given link, participants were asked to provide sociodemographic information, including age, education level, gender, and professional status (active vs. non-active). This process was repeated each time they entered a new task or difficulty level, allowing them to freely choose which activities/levels to engage with. In the specific case of the Multiple Sclerosis group, means (age, education) and proportions (gender and status) were inserted for convenience, with individual information collected in-person. Following this, they received task-specific instructions and completed two practice trials, with feedback and explanations provided in case of incorrect answers. Subsequently, participants answered the multiple-choice questions that comprised the block (variable number of questions, ranging from 10 to 48) and received positive or negative feedback after each response. At the end of each task (block of questions), participants were informed of their overall performance, namely their hit rates and average response time. Based on their performance levels, they were advised whether to increase the difficulty level. Before leaving the block, participants rated the difficulty of the task using a 5-point scale (1 = very easy; 5 = very difficult) and the adequacy of the instructions using a 3-point scale (1 = inadequate/hard to understand; 3 = adequate/easy to understand). They were also asked whether they had received any guidance in choosing the correct responses and if they had encountered any technical issues while using the computer (both questions had yes/no response options).

For CQ users, they were simply invited to play and could access the platform using a computer, tablet, or smartphone. Unlike Cerup, the CQ platform could be used anonymously, with or without an account. Users with an account (i.e., those with a username and password) provided sociodemographic information (e.g., age, gender) only if they chose to do so. The eight activities were organized as shown in Figure 1, with a simpler layout and a different structure compared to Cerup. Upon selecting a specific activity, users could choose to play at the easy, medium, or difficult level. Unlike Cerup, which featured a fixed set of questions created by the authors, CQ was constantly updated with new questions, created by other users. These questions were retrospectively classified by the administrators based on predefined criteria (see above). Each block consisted of eight questions, randomly selected by the platform for each new game. The feedback procedure was similar to that of Cerup. Users were not required to answer any additional questions beyond the activity itself. The data analyzed in this study correspond to registered users’ activity between October 2023 and September 2024.

### 2.4. Analysis

First, we analyzed the number of completed blocks (productivity) per platform (2 levels: Cerup vs. CQ), user-type (5 levels, individuals vs. 4 group types), and activity-related variables activity (8 levels, W1–W4 and S1–S4), complexity (4 levels: L1–L4), and type (2 levels: word vs. sentence). Though our primary focus was on activity-related factors, platform differences were examined to see whether variations in layout, block size (smaller in CQ), and context (Cerup being part of a usability study, whereas CQ was not) affected users’ engagement and performance. User type was investigated for exploratory purposes. Performance accuracy was analyzed in similar terms. To that end, we used linear mixed-effects models (lme4 package Version 1.1-37, [55]) which are more suitable for unbalanced data (e.g., variable number of responses per platform when computing accuracy). Each entry in the database corresponded to a user-account x activity. If, e.g., user-account A01 had participation records in two activities, then it would contribute with two entries. For Cerup, participants did not have an account but provided sociodemographic data every time they completed one block. By matching this information across blocks, we were able to trace the activity per user. Fixed factors (user type, platform, and activity-related variables) were tested individually in separate models, with entries (user-account x activity) included as random intercepts. In these models, effects are presented as comparisons against a reference level. While two-level factors give rise to a single comparison, factors with multiple levels (user type, activity, complexity) required the definition of a baseline, reference level - which we chose to be the one with the lowest mean. Additionally, we performed model comparisons to determine which activity-related factor best fit the data, using the Bayesian information criterion (BIC), which is recommended for selecting simpler models.

Participants’ spontaneous engagement with the platforms was assessed based on the variety of activities explored and patterns of activity co-occurrence per user. Variety was analyzed descriptively: we determined the number of different activities each user tried (integer from 1 to 8, with 8 indicating maximum variety) and described the distribution of these 8 levels (how many accounts explored a maximum of 1, 2, etc. activities). Co-occurrence patterns referred to the activities that tended to be played by the same user (e.g., users that play W1 also tend to play activities X and/or Y9). These patterns were examined using network analysis based on high-dimensional undirected graph estimation [56] and EBIC (extended Bayesian information criterion), as implemented in JASP. Graph analyses provide, among other measures, indices of the strength of co-occurence (weights) among several types of events (nodes; here, activities) and provide concise graphical representations of the whole system of interrelations.

Participants’ perceptions of difficulty and adequacy of instructions—data obtained from the Cerup platform—were analyzed separately. Perceived difficulty was assessed using one-sample *t*-tests against chance levels (50%), comparing activity type (word vs. sentence level) and activity (8 tasks). Instruction clarity was analyzed for each task. Finally, we analyzed the correlations between accuracy, perceived difficulty, and instruction clarity. Given violations of normality in the data distributions, we applied non-parametric correlation analyses.

## 3. Results

### 3.1. Productivity

Participants completed a total of 271 blocks of questions. As shown in Table 3, institutional accounts provided 81% of the responses, indicating that the group mode (i.e., supervised) prevailed. Among institutional users, individuals with MS were the most productive, completing 45 blocks per user (notably, there was only one user in this category).

The platform showed no significant effects (*p* > 0.65). Concerning differences among the eight activities, W1 (*B* = 1.81, *CI* = 0.29–3.33, *p* = 0.020) and W2 (*B* = 1.61, *CI* = 0.01–3.21, *p* = 0.048) elicited significantly more responses than S4, which had the lowest adherence. Word- and sentence-level activities resulted in a comparable number of completed blocks (*p* = 0.096), and complexity had no significant effect on productivity (*ps* > 0.37). Among user types, individual users, who had the lowest mean number of responses per user, underperformed significantly all other user types (*ps* < 0.026), except for nursing home residents (*p* > 0.86).

### 3.2. Preliminary Usability Data (Cerup)

The adequacy of instructions was rated between 2 and 3 on a 3-point scale for all tasks. One-sample *t*-tests showed that mean ratings were significantly above the midpoint of 2 (word-level: *t*(28) = 6.77, *p* < 0.001, *d* = 1.26; sentence-level: *t*(26) = 7.32, *p* < 0.001, *d* = 1.41). Regarding perceived difficulty (scale 1–5), word-level tasks received ratings that did not significantly differ from the midpoint of 3 (neither hard nor easy, *p* > 0.14). However, sentence-level tasks were rated significantly below the mid-point, indicating ease (*t*(26) = −2.29, *p* < 0.030, *d* = −0.44).

Accuracy was strongly negatively correlated with perceived difficulty (*ρ* = −0.541, *p* < 0.001) and positively with adequacy of instructions (*ρ* = 0.532, *p* < 0.001). However, no significant association was found between perceived difficulty and adequacy of instructions (*p* > 0.61).

### 3.3. Accuracy

**Figure 2:**
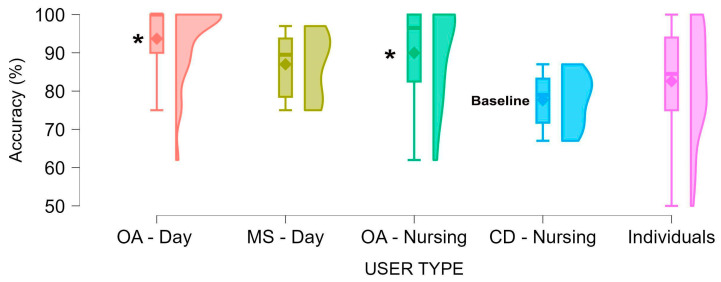
Accuracy by user type. OA—older adults; MS—multiple sclerosis; CD—cognitive deficit; day—day center; nursing—nursing home. All user types other than individuals participated in group sessions. Asterisks denote significantly higher performance levels relative to the lowest mean baseline.

Accuracy was consistent across platforms (*p* = 0.504). Compared to individuals with cognitive deficits (CD-nursing, who had the lowest mean accuracy), day centers (*B* = 15.73, *CI* = 7.10–24.35, *p* < 0.001), and nursing homes (*B* = 11.93, *CI* = 2.24–21.63, *p* = 0.016) demonstrated significantly better performance (Figure 2). Individual users (*p* = 0.305) and MS patients did not significantly differ from the CD-nursing baseline, though the latter exhibited marginally better performance (*B* = 9.28, *CI* = 1.66–20.22, *p* = 0.096).

**Figure 3:**
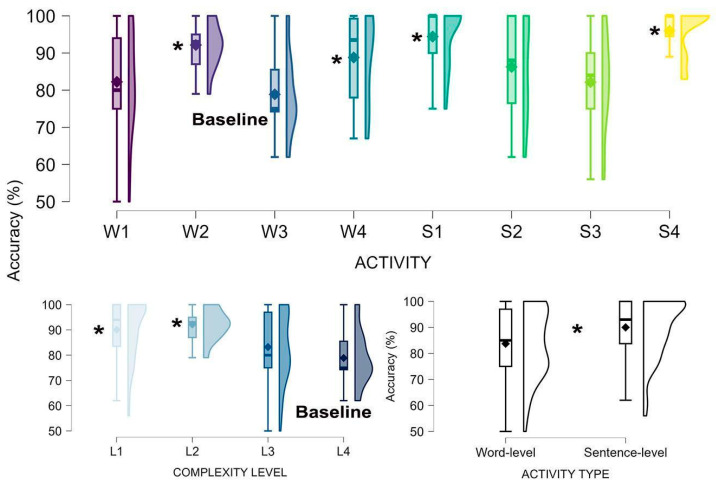
Accuracy as a function of activity (8 levels), complexity scores (4 levels), and type (word- vs. sentence-level activity). Asterisks denote significantly higher performance levels relative to the lowest mean baseline.

Figure 3 depicts accuracy across activity-related factors. Compared to the baseline (W3, which had the lowest mean accuracy), performance was significantly higher in S1 (B = 16.69, CI = 8.76–22.63, *p* < 0.001), W2 (B = 14.16, CI = 5.97–22.35, *p* = 0.001), W4 (B = 10.26, CI = 1.68–18.84, *p* = 0.019), and S4 (B = 15.13, CI = 5.73–24.53, *p* = 0.002). Word-level activities resulted in lower accuracy than sentence-level ones (B = −5.92, CI = −10.24–1.60, *p* = 0.007). Regarding complexity, L1 (B = 11.45, CI = 13.82–19.09, *p* = 0.003) and L2 (B = 14.13, CI = 5.16–23.09, *p* = 0.002) outperformed L4 (which had the lowest mean accuracy), while L3 did not significantly differ from L4 (*p* = 0.24). Comparisons of the three models based on BIC indicated that the model using activity as a predictor had a poorer fit compared to the other two models (activity type, χ^2^(4) = 14.38, *p* = 0.006; complexity, χ^2^(6) = 23.24, *p* = 0.007). Among the latter two, the model incorporating activity type demonstrated a significant advantage (χ^2^(2) = 8.85, *p* = 0.012).

### 3.4. Spontaneous Navigation Patterns

#### 3.4.1. Variety per User

**Figure 4:**
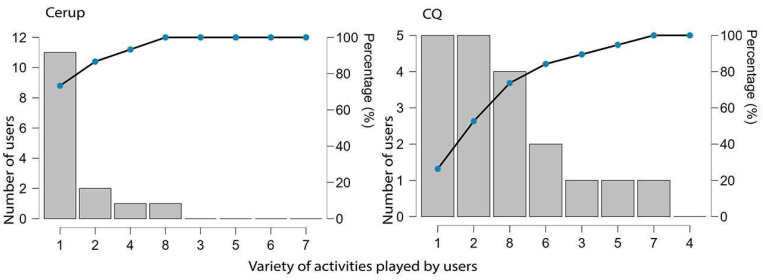
Distribution of users according to the variety of different activities engaged in.

Users (*n* = 34) could attempt up to eight different activities; however, most did not engage in the full range. As shown in Figure 4, 53% of Cerup users participated in only one or two activities (Md = 1, Mode = 1 different activities), while in CQ, this percentage increased to 87% (Md = 1, Mode = 1).

#### 3.4.2. Activity Co-Occurrence

**Figure 5:**
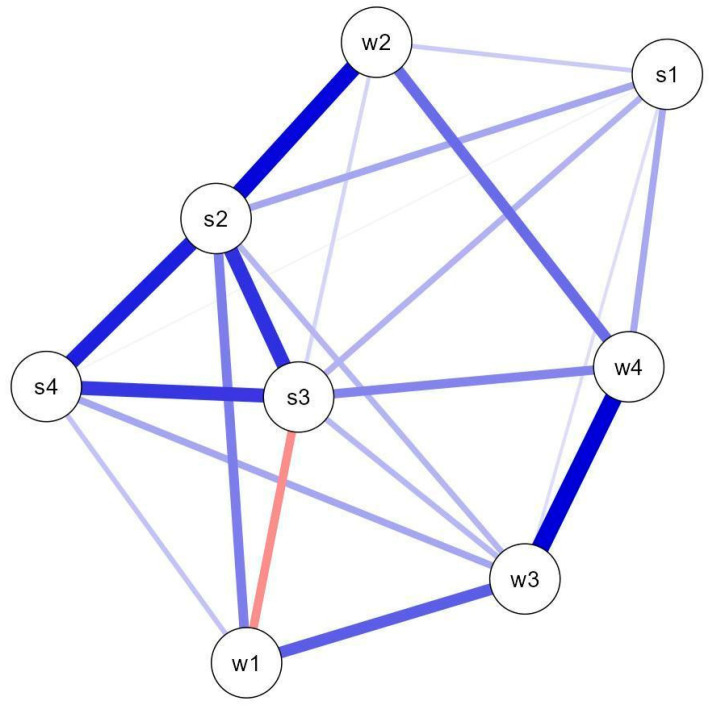
Network plot illustrating the co-occurrence patterns (connecting lines) among the eight different activities, here represented as circles. Thicker lines represent increased weights (increased frequency of co-occurrence). Red lines indicate negative associations; here, the more participants play activity W1, the less they play S3 and vice-versa.

Figure 5 illustrates the associations between activities based on the number of blocks completed (including zero). The network analysis output indicated a densely connected structure (sparsity = 0.214, 22/28 non-zero edges), suggesting multiple activity co-occurrence patterns across users. The strongest connections occurred among S2–S3–S4 (weights: S2–S3 = 0.303; S2–S4 = 0.327; S3–S4 = 0.294), S2–W2 (0.359), and W3–W4 (0.378).

## 4. Discussion

In this study, we describe the process of developing evidence-based adaptive guidance for task selection in two online platforms for semantic-based cognitive stimulation: Cerup and CQ. The guidance we aimed to create consisted of a set of optimal paths—groups of activities sharing a common semantic target—ordered by increasing levels of challenge based on performance metrics. These structured paths could allow users (both players and/or supervisors) not only to benefit from a convergent context, i.e., maintaining focus on a single target due to activity grouping, but also choose their preferred level of challenge. To validate the task-related adaptive guidance, we tested two hypotheses: First, we hypothesized that the eight activities provide different levels of challenge, justifying the creation of optimal paths that progress from less to more challenging tasks. Second, we proposed that in the absence of guidance, the platforms’ current layout allows or even encourages spontaneous activity sequences (real paths) that diverge from the optimal paths. Both hypotheses received empirical support: performance accuracy was influenced by the task itself, its predefined level of complexity, and whether it engaged with word- vs. sentence-level semantics. Concerning real vs. optimal paths, we observed a clear dissociation: real paths presented multiple patterns, only and partially explained by the spatial contiguity of activities in the main menu. Notably, in the current platform layout, this spatial organization has little alignment with activity groupings and challenge-related hierarchies.

### 4.1. Real vs. Optimal Navigation Paths

In the preliminary usability data (Cerup study), the instructions were found to be sufficiently clear, with perceived difficulty levels ranging from medium (word-level activities) to low (sentence-level). Accuracy, analyzed for both Cerup and CQ, was equivalent across platforms and high overall. This is evident in Figure 4 and Figure 5, where interquartile values exceed 50% and mean values surpass 70%. These results indicate satisfactory performance and positive user experience. For Cerup, accuracy strongly correlated with both perceived difficulty and clarity of instructions; however, the latter two did not correlate with each other. This suggests that variations in user difficulty levels were not solely due to instructions but rather reflected the inherent requirements of each activity. The observation that word-level activities were perceived as more difficult than sentence-level ones—mirroring performance accuracy patterns—reinforces this conclusion. This task-specific user experience and performance level from users emphasizes the appropriateness of guidance based on an optimal navigation path.

Regarding spontaneous navigation (i.e., the actual user paths), participants exhibited a relatively disorganized choice of activities, displaying multiple patterns of activity co-occurrence (dense network). Despite this, the most frequent patterns aligned with the spatial contiguity principle: the strongest co-occurrences—groups S2–S3–S4, S2–W2, and W3–W4—comprised activities that were adjacent either vertically or horizontally (see Figure 1). Productivity measures per task also reflected this principle, since W1 and W2—vertically adjacent in CQ and horizontally in Cerup—were the highest number of responses per user. The positioning of W1 stands at the top-left position of the 4 × 2/2 × 4 panel (CQ/Cerup, see Figure 1), suggesting that users may have navigated the menu in a text-like manner, from top to bottom and left to right. Finally, activity diversity was low, with participants often focusing on just one or two types of activities.

Accuracy data showed that users’ task selection (apparently guided by spatial contiguity) did not align well with their likelihood of success (task-dependent accuracy in performance). Specifically, the two most frequently attempted activities (W1 and W2, significantly higher than the least selected, S2), excluded three of the four activities with the highest accuracy rates (S1, W2, W4, S4, significantly higher than the most challenging one, W3). Examining complexity levels (four levels, some encompassing different activities), we found that accuracy decreased with complexity: the two lowest-complexity levels (L1–W4, S1, S3–S4, L2–W2) elicited significantly better performance than the highest level (L4–W3), while L3 (W1, S2) did not differ from L4. The hypothesized difference between word- and sentence-level activities in performance accuracy was also supported by the results, with the latter type generating higher success rates. Theoretically, this may indicate a beneficial influence of semantic context at the sentence-level. Practically, it suggests that beginning with sentence-level activities may also be beneficial to users. Overall, these findings provide validation to our theoretically driven word- vs. sentence-level categorization and pre-assigned complexity scores, supporting an optimal path based on a progression from less to more challenging activities.

We also considered two additional, task-independent influences on navigation and performance—user type and platform (Cerup vs. CQ)—both of which were by-products of the data collection method and not part of our main focus. Regarding user type, individual users were less productive and less accurate in their responses compared to all other groups, except for older adults in nursing homes. One possible explanation is that the increased supervision characterizing group sessions provides structure, support, and opportunities for social interaction and competition, which may enhance motivation and engagement. However, alternative explanations are possible. For instance, since participants’ cognitive abilities were not assessed, we cannot rule out the possibility that individual users had lower cognitive aptitude. That stated, the fact these individuals successfully used computers, tablets, or smartphones to engage in the study suggests otherwise. Additionally, our proposed supervision–socialization hypothesis aligns with the poorer performance observed among older adults in nursing homes, where staff members/technicians are often overwhelmed with various tasks and responsibilities, unlike those in day centers or smaller nursing homes specializing in cognitive deficits, which participated in this study. As for platform differences, although Cerup and CQ featured the same activities, their starting menu layouts differed. Despite this, the impact of platform layout on navigation patterns and accuracy was negligible, suggesting that neither layout was inherently more preferable to the other.

In summary, participants may have made suboptimal choices in activity selection. This could be due to their failure to follow a “start-small” path from the least to the most challenging activities, selecting tasks that were too easy for their skill level, or not leveraging cross-task commonalities regarding target domains. Of course, this is not a shortcoming on the part of the participants, since identifying these relationships independently would be nearly impossible. Nevertheless, these findings highlight the importance of adaptive guidance in task selection.

### 4.2. Adaptive Guidance for Task Selection Based on Two Optimal Paths

We conceptualized optimal paths as ideal sequences of activities that users should follow to benefit from grouping activities with similar targets (principle of convergence) and/or progressing from less to more challenging tasks (principle of incrementally). However, it should be noted that optimal paths represent the recommended approach only if the goal is to ensure a smooth experience in terms of focus and challenge. Some users may have different objectives, such as seeking maximum challenge, focusing on a specific domain (e.g., categorization), or a combination of these (high or low challenge within a specific domain). Therefore, optimal paths should not be viewed as rigid recommendations but rather as flexible maps/guides that provide useful elements for users and supervisors to make informed decisions based on users’ characteristics and/or diagnostic performance.

**Figure 6:**
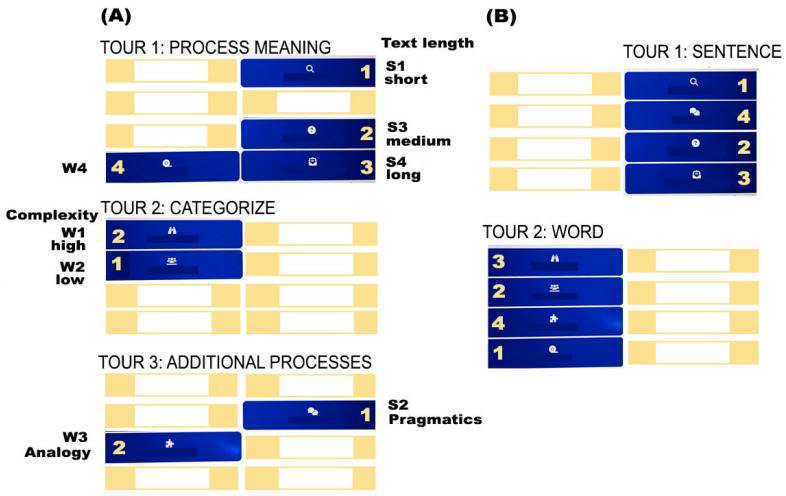
Two evidence-based maps for optimizing navigation within the Cerup/CQ platforms: **Path** (**A**) is based on task complexity (both within- and between-tour sequencing) and target domains (the tours themselves), while **Path** (**B**) is based on the semantic unit (sentence- vs. word-level activities, the tours) and task complexity. Numbers indicate optimal task order.

Based on our a priori structural analysis of activities, combined with empirical findings, we identified two optimal paths (Figure 6): Path A groups activities into three domains, or “tours” (meaning extraction, categorization, and extra-semantic processes, Figure 6A), with activities ordered by increasing complexity within each tour. Path B comprises two groups (tours) based on sentence- vs. word-level semantics (Figure 6B), with activities similarly ranked by complexity. In both cases, the principle of incrementality is embedded in the recommended sequence of tours: in Path A, Tour 3 is expected to be the most challenging; in Path B, the sentence-level tour is suggested as the starting point. Again, these recommendations depend on the goals at stake, as they may be less relevant when the objective is to focus on a given target domain within semantics.

The incorporation of task complexity into these two maps requires some considerations, highlighting the need for further refinement. We assigned complexity scores to each of the eight tasks, based solely on the structural analysis of activities. These scores were empirically validated through two accuracy predictor types: one based on individual activity (eight levels) and another that grouped activities with the same score into four complexity levels. The latter model, which provided a better fit, classified S3 as a level 1 (easiest) activity, alongside S1 and S4. Consequently, we positioned S3 between S1 and S4, ordering them according to text size (sentence, sequence of sentences, discourse). However, in the model with the eight distinct activities as predictors, S3 emerged as more challenging than initially expected, comparable to level 3 and 4 activities (S2 and W3). This discrepancy likely resulted from additional cognitive demands at medium and difficult levels that were not accounted for in the initial complexity scores. Further research should explore this issue through a more refined analysis that considers within-task difficulty as a factor. Until then, we recommend that users test whether the current ordering is effective or whether S3 should be positioned at a higher difficulty level (e.g., sequencing S1, S4, and then S3 within the tour).

Beyond task selection, the context of administration may also be relevant in optimizing Cerup/CQ experience. Group-based participation has been shown to enhance adherence and productivity; therefore, the impact of group vs. individual modalities should be considered for each context. For example, starting in a group setting may be beneficial for users with low motivation.

## 5. Conclusions

Our study suggests that viewing a specific cognitive stimulation program (BOX, adapted to Cerup/CQ) as a bundle of complementary activities may not be the most effective approach. Rather than merely complementing each other, different activities may represent varying levels of challenge. Given that users are unlikely to recognize these differences—our findings indicate that they do not—it is reasonable to provide adaptive guidance for task selection. We addressed this need by defining optimal paths, or ways to navigate the different activities that facilitate progression from lowest to highest challenge while also grouping activities based on similar targets. While our proposal requires further validation—specifically, by comparing performance between users following the optimal paths with those without guided navigation—the method presented here, which is largely evidence-based, may serve as a foundation for refining adaptive guidance in other programs with structurally distinct activities addressing the same domain. Future research should explore additional methods in order to define the optimal paths for cognitive rehabilitation. For example, artificial intelligence or algorithms could be added to match performance to the number of examples, paths, or task bundles. Additionally, stratified sampling could be employed to indicate possible variations in cognitive impairment levels. Also, future comparisons between facilitator-led versus individual task completion modes may reveal important differences in engagement or learning outcomes. The exploration of technology-driven methods could build on current findings and help us better design effective pathways for cognitive tasks).

## Figures and Tables

**Table 1:** Number of users with an account that were actively engaged with the Cerup and CQ platforms.

	Group Accounts				Total	Individual Accounts	Total
	OA—Day	MS—Day	OA—Nursing	CD—Nursing			
Cerup	--	1	--	--	1	14	15
CQ	7	--	5	1	13	6	19
	14					20	34

Note: OA—older adults; MS—multiple sclerosis; CD—cognitive deficit; day—day center; nursing—nursing home.

**Table 2:** Complexity indices per activity.

	W1	W2	W3	W4	S1	S2	S3	S4
Single vs. composite	1	1	2	1	1	1	1	2
Number of steps	2	1	3	1	1	2	1	1
ACP	1	1	1	0	0	1	0	0
Uncertainty in ACP	1	0	1	-	-	1	-	-
Complexity index (sum)	5	3	7	2	2	5	2	2

Note: ACP—additional cognitive processes.

**Table 3:** Number of blocks completed by platform, user type, activity, activity type and activity complexity.

Variable	Blocks	%	Mean per User	Variable	Blocks	%	Mean per User
Platform				Activity			
Cerup	68	25	4.5	W1 *	69	25	2.0
CQ	203	75	10.7	W2 *	44	16	1.3
				W3	30	11	0.9
User type				W4	28	10	0.8
OA-day *	116	43	16.6	S1	34	13	1
MS-day *	45	17	45	S2	20	8	0.6
OA-nursing	16	6	3.2	S3	28	10	0.8
CD-nursing *	42	15	42	S4 (Ref)	18	7	0.5
Individuals (Ref)	52	19	2.7				
				Activity complexity			
Activity type				L1 *	108	40	3.2
Word	134	49	3.9	L2 *	44	16	1.3
Sentence	137	51	4.0	L3	89	33	2.6
				L4 (Ref)	30	11	0.9

Note: OA—older adults; MS—multiple sclerosis; CD—cognitive deficit; day—day center; nursing—nursing home; L1–L4—level 1–4; Ref—variable level showing the lowest values, against which the other levels were compared; Asterisks denote significantly higher values compared to the reference level.

## Data Availability

The original data presented in this study are openly available in “Linguistic semantic online exercises as cognitive stimulation tools” at Supplementary Materials.

## Supplementary Materials

The following supporting information can be downloaded at: https://osf.io/a3y7s/; 18 April 2025; Questions in Portuguese.
